# Thematic Issue: Protists

**DOI:** 10.1111/emi.12203

**Published:** 2013-07-23

**Authors:** Matthew S Twigg, Karen Tait, Paul Williams, Steve Atkinson, Miguel Cámara

**Affiliations:** 1 School of Molecular Medical Sciences, Centre for Biomedical Sciences, University of NottinghamNG7 2RD, Nottingham, UK; 2 Plymouth Marine LaboratoryProspect Place, Plymouth, PL1 3DH, UK

## Abstract

*Ulva* zoospores preferentially settle on *N*-acylhomoserine lactone (AHL) producing marine bacterial biofilms. To investigate whether AHL signal molecules also affect the success and rate of zoospore germination in addition to zoospore attraction, the epiphytic bacteria associated with mature *Ulva linza* were characterized and bacterial isolates representative of this community tested for the ability to produce AHLs. Two of these AHL-producing isolates, *Sulfitobacter* spp. 376 and *Shewanella* spp. 79, were transformed with plasmids expressing the *Bacillus* spp. AHL lactonase gene *aiiA* to generate AHL-deficient variants. The germination and growth of *U. linza* zoospores was studied in the presence of these AHL-deficient strains and their AHL-producing counterparts. This revealed that the AHLs produced by *Sulfitobacter* spp. and *Shewanella* spp. or the bacterial products they regulate have a negative impact on both zoospore germination and the early growth of the *Ulva* germling. Further experiments with *Escherichia coli* biofilms expressing recombinant AHL synthases and synthetic AHLs provide data to demonstrate that zoospores germinated and grown in the absence of AHLs were significantly longer than those germinated in the presence of AHLs. These results reveal an additional role for AHLs *per se* in the interactive relationships between marine bacteria and *Ulva* zoospores.

## Introduction

The green seaweed *Ulva* is the most common macro-alga contributing to biofouling of man-made surfaces throughout the world, mainly due to its tolerance to diverse environmental conditions and antifouling surface coatings, and also to its enormous reproductive potential, with vast quantities of motile zoospores (or zygotes from the fusion of gametes) being released from each thallus (Callow *et al*., 1997). Once released from the thallus, the swimming zoospores select surfaces for attachment by ‘sensing’ a surface, and this is followed by temporary adhesion. If conditions are unsatisfactory, the zoospore detaches and continues to search for an optimum location. Once conditions are ‘sensed’ as satisfactory, the zoospore excretes a glycoprotein adhesive to form a permanent attachment, differentiates and grows to form a mature alga (Callow *et al*., 2000a). Several factors are thought to influence the surface selection of *Ulva* zoospores, including negative phototaxis, surface chemistry, wettability, surface topography and also the presence of a bacterial biofilm (Callow and Callow, 2000; Joint *et al*., 2000; Callow *et al*., 2000b; 2002). Zoospores were shown to preferentially settle on top of bacteria, suggesting a direct interaction between the bacteria and zoospores (Joint *et al*., 2000). *Ulva* zoospores can also utilize bacterial quorum-sensing (QS) signal molecules, specifically the *N*-acylhomoserine lactones (AHLs) as a cue for the selection of sites for attachment (Joint *et al*., 2002; Tait *et al*., 2005; 2009). QS molecules such as the AHLs are used by bacteria to coordinate their behaviour at the population level and by collectively controlling expression of multiple genes including those involved in secondary metabolism, virulence, motility and biofilm development (Williams *et al*., 2007). AHLs affect swimming behaviour of the zoospores through a process of chemokinesis (Wheeler *et al*., 2006), and the detection of AHLs by zoospores causes an increase in intracellular Ca^2+^ levels (Joint *et al*., 2007).

In addition to their effect on *Ulva* zoospore settlement, bacteria appear to have a profound impact on the morphology and growth of *Ulva*. When grown axenically, *Ulva lactuca* adopts an atypical ‘pin cushion’ undifferentiated morphology as opposed to the normal foliaceous growth developed when associated with marine bacteria (Provasoli and Pintner, 1980). Such changes in morphology, when grown axenically, have also been observed in *Monostroma oxyspermum* (Tatewaki *et al*., 1983; Matsuo *et al*., 2003), *Ulva linza*, *Ulva pertusa* and *Ulva compressa* (Nakanishi *et al*., 1996; Marshall *et al*., 2006). Adding back bacterial strains to axenic *Ulva* cultures has been shown to restore the foliaceous morphology (Nakanishi *et al*., 1996; Marshall *et al*., 2006). Of 1555 bacterial strains isolated from *U. pertusa*, 676 strains (41%) showed morphogenesis activity (Nakanishi *et al*., 1996). For *U. linza*, 13 out of 20 unique strains isolated from the algae induced morphological change when added to axenic cultures, and five of those also increased the relative growth rate of the alga (Marshall *et al*., 2006). However, phylogenetic analysis of these strains indicated that the bacterial effect on *Ulva* was independent of bacterial phylogeny: members of the *Proteobacteria*, *Bacteroidetes* and Gram-positive cocci all positively impacted *Ulva* growth (Marshall *et al*., 2006).

This suggests that there are either multiple cues produced by epiphytic bacteria that stimulate growth and morphogenesis of *Ulva* or that a common metabolite, produced by a wide range of bacteria, is responsible for these effects. There are reports of marine bacteria producing plant growth regulators and vitamins which may affect the morphological differentiation of algae (Maruyama *et al*., 1986; Croft *et al*., 2006). For example, an algal morphogenesis inducer, named thallusin, has been isolated from a *Bacteroidetes* strain. Very low concentrations of this compound strongly induced differentiation in *M. oxyspermum* (Matsuo *et al*., 2005). Additionally, other algal growth hormones such as cytokinin-type hormones, auxin-type hormones and indole-3-acetic acid have been shown to be produced by marine bacteria (Maruyama *et al*., 1986; Bradley, 1991). It has been hypothesized that the effect of bacteria on algal growth and morphology may be due to: (i) bacteria being responsible for supplying nitrogen to algae, a suggestion based upon an observation that bacterial strains isolated from green alga possess the nitrogenase gene *nifH*, and/or (ii) that some bacterial species metabolize plant hormones (Chisholm *et al*., 1996; Ashen and Goff, 2000).

Although AHLs affect *Ulva* zoospore settlement, their potential influence on the later stages of *Ulva* growth and development has never been investigated. Hence, the aim of the present study was to explore whether AHL signal molecules from marine bacteria could influence *Ulva* germination and growth. The bacterial population associated with *U. linza* was first phylogenetically characterized, bacteria representative of the epiphytic community isolated and their AHL profiles characterized using AHL bioreporters and liquid-chromatography tandem mass spectrometry (LC-MS/MS). The germination and growth response of *Ulva* zoospores exposed to bacterial biofilms composed of AHL-producing strains indigenous to the *Ulva* bacterial population was compared with the response to the same strains transformed with plasmids expressing the recombinant *Bacillus* sp. lactonase enzyme AiiA to create AHL-deficient variants (Dong *et al*., 2002). Additionally, *Ulva* germination and germling growth was measured in response biofilms of transgenic *Escherichia coli* which expressed various AHL synthase genes and with synthetic AHLs alone.

## Results

### Impact of AHL-producing biofilms from *Ulva* endogenous bacteria on zoospore germination

A phylogenetic tree resulting from the alignment and analysis of a clone library composed of 76 individual 16S rDNA clones at 97% sequence similarity shows the *U. linza* thallus epiphytic bacterial community to be dominated by *Alphaproteobacteria*, *Gammaproteobacteria*, *Flavobacteria* and *Sphingobacteria* (Fig. S1). Additionally, signal molecule characterization using both *E. coli lux*-based bioreporters and LC-MS/MS showed bacterial strains isolated from *Ulva* to produce a wide variety of AHLs (Table S1). The 16S rDNA clone library of bacteria associated with *Ulva* showed both *Sulfitobacter* (*Alphaproteobacteria*) and uncultured *Alteromonadales* (*Gammaproteobacteria*) to be abundant within the population. Based on these findings, two AHL-producing strains from these groups of bacteria were selected in order to investigate the zoospore response to *Ulva*'s indigenous bacteria. To compare the impact on germination of these strains with their AHL-deficient counterparts, *Sulfitobacter* spp. 376 and *Shewanella* spp. 79 were transformed with pMT01, a plasmid harbouring the *aiiA* AHL lactonase gene from *Bacillus* sp. 240B1 within the broad host vector pBBRIMCS-5 (Kovach *et al*., 1995). Successful transformation with the pMT01 plasmid rendered the cognate AHLs of 376 and 79 biologically inactive as AiiA hydrolyses the homoserine lactone ring (Dong *et al*., 2000). Inactivation of AHL production by AiiA was confirmed by the lack of activation of appropriate *lux*-based *E. coli* AHL bioreporters with solvent extracts from cell-free supernatants of these strains (Fig. S2).

**Figure 1:**
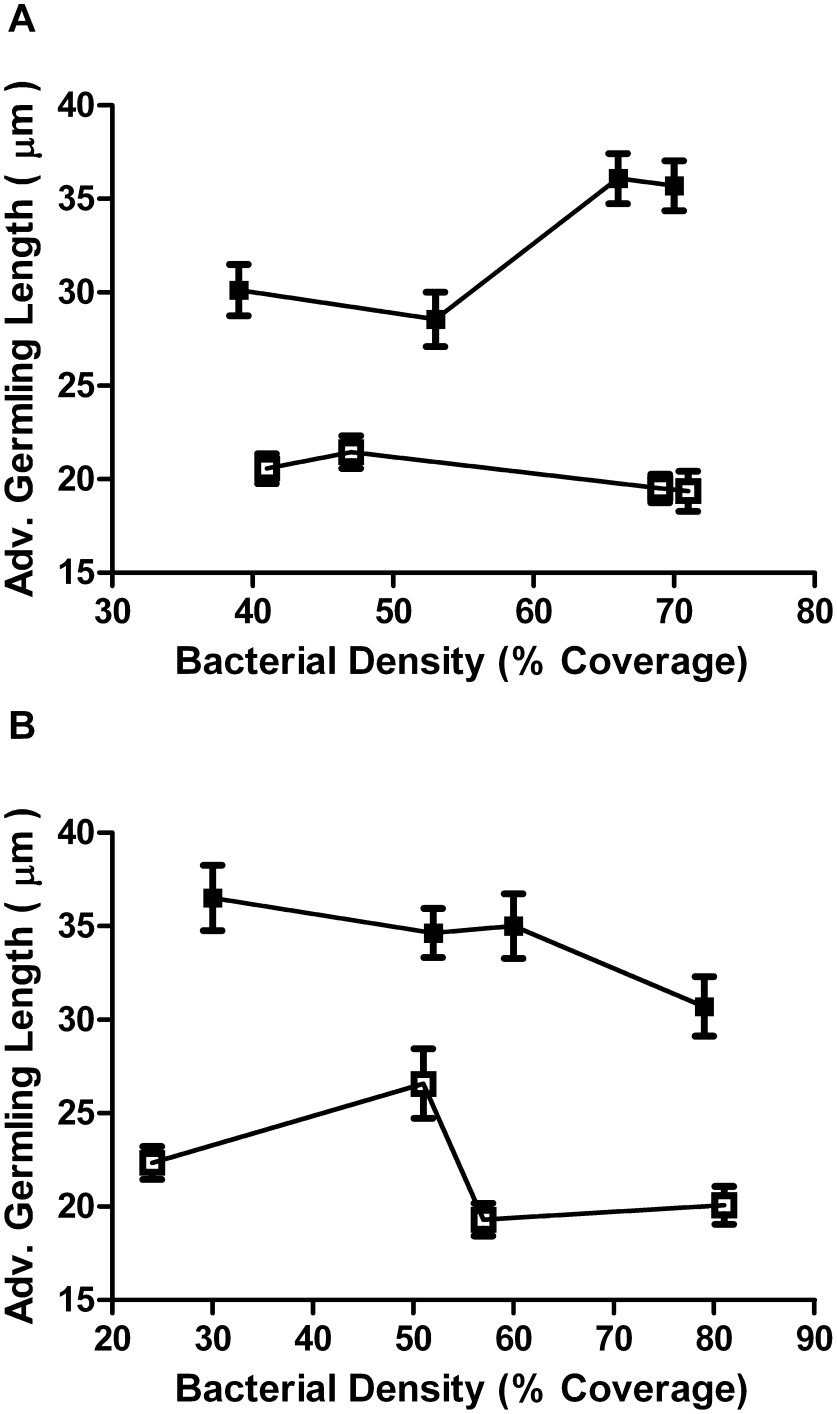
Effect of marine bacterial biofilms on Ulva zoospore germling growth.
AAverage germling length of zoospores exposed to biofilms of AHL-expressing *Shewanella sp.* 79 pBBRIMCS (□) and non-AHL expressing *Shewanella sp.* 79 pMT01 (□) at 48 h incubation.BAverage germling length of zoospores exposed to biofilms of AHL expressing *Sulfitobacter sp.* 376 pBBRIMCS (□) and non-AHL expressing *Sulfitobacter sp.* 376 pMT01 (□) at 48 incubation. Error bars represent 95% confidence intervals.

*Ulva* zoospores were settled onto *Shewanella* spp. 79 pBBRIMCS, *Shewanella* spp. 79 pMT01, *Sulfitobacter* spp. 376 pBBRIMCS and *Sulfitobacter* spp. 376 pMT01 biofilms of varying bacterial density. Zoospore germination and average germling length was measured after 48 h incubation (for representative images of germinated zoospores exposed to AHL-producing and non-producing marine bacterial biofilms, see Fig. S3). The varying volumes of inoculum used to grow biofilms of both strains produced biofilms with bacterial densities ranging from approximately 20–75% coverage, with no major difference in biofilm density observed between strains carrying the pBBRIMCS or pMT01 plasmids. After 48 h incubation, average germling length was greatly increased for zoospores settled on biofilms of both *aiiA*-expressing, AHL-deficient *Shewanella* spp. and *Sulfitobacter* spp. strains compared with the AHL-producing biofilms (Fig. 1). Additionally, successful zoospore germination was significantly increased on the AHL-deficient *Shewanella* spp. 79 pMT01 biofilms with an average percentage of germination of 77.9% (± 6.4%) in comparison with 53.4% (± 7.2%) on AHL-producing *Shewanella* spp. 79 pBBRIMCS biofilms. This trend was also observed in *Sulfitobacter* spp. 376 where the percentage of germination on *Sulfitobacter* spp. 376 pMT01 biofilms was 80.4% (± 2.8%) in comparison with 62.4% (± 6.3%) on *Sulfitobacter* spp. 376 pBBRIMCS biofilms.

### *Ulva* zoospore germination response when exposed to biofilms of *E. coli* expressing recombinant AHL synthases

**Figure 2:**
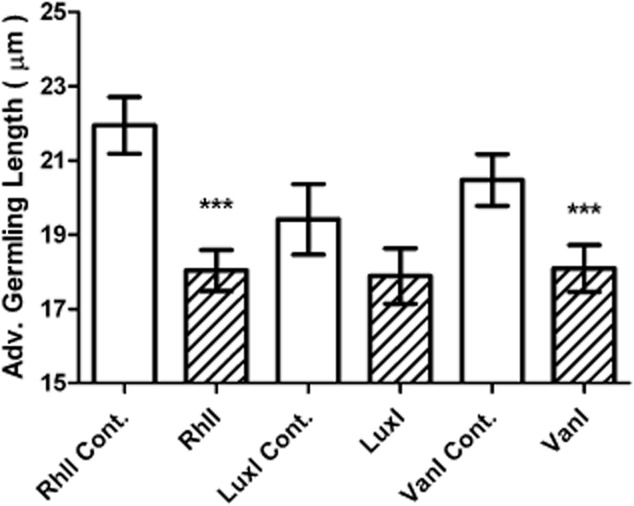
Effect of recombinant *E. coli* biofilms on *Ulva* germling growth. Average *Ulva* germling length when exposed to biofilms of *E. coli* producing AHLs from plasmids expressing recombinant AHL synthases, in comparison with those from their *E. coli* counterparts harbouring the corresponding control vectors without the AHL syntheses after 24 h incubation. The plasmids used in this experiment are listed in Table S2. Error bars represent 95% confidence intervals and asterisks show those values significantly different to the controls (one-way ANOVA * = *P* ≤ 0.001).

The changes in zoospore germination observed following the expression of *aiiA* in the AHL-producing *Sulfitobacter* spp. 376 and *Shewanella* spp. 79 strains may be a consequence of either the direct action of AHLs on the zoospores or indirectly due to the loss of an AHL-dependent bacterial phenotype, or the action of the lactonase. To distinguish between these possibilities, *Ulva* zoospore germination was assayed using biofilms composed of transgenic *E. coli* strains that expressed different recombinant AHL synthase genes. These synthases were RhlI from *Pseudomonas aeruginosa* (producing C4-HSL), LuxI from *Vibrio fischeri* (producing 3-oxo-C6-HSL) and VanI from *V. anguillarum* (producing 3-oxo-C10-HSL) (Latifi *et al*., 1995; Milton *et al*., 1997; Joint *et al*., 2002). All recombinant AHL synthase genes were expressed from plasmids within the *E. coli* host and compared with *E. coli* harbouring the respective vector plasmids without recombinant AHL synthase genes (see Table S2). These strains constitutively produce AHLs, resulting in a more stable assay for monitoring the response of *Ulva* zoospores to AHLs (Joint *et al*., 2002). Owing to the detrimental effect of the osmotic pressure of seawater on the growth and survival of *E. coli* biofilms, zoospore slides were incubated for a reduced time of 24 h in 70% sterile filtered seawater prior to being fixed and stained. At 24 h incubation, there was a small yet significant decrease in average germling length when *Ulva* zoospores were settled and grown on biofilms of *E. coli* expressing *rhlI* and *vanI*. Although the response to *E. coli* expressing *luxI* was not found to be significant, a modest decrease in the presence of this biofilm was also apparent (Fig. 2). Reduction in successful zoospore germination was observed for all AHL-producing biofilms of *E. coli* strains at 24 h in comparison to their respective AHL-deficient vector control stains (33% for RhlI, 19% for LuxI and 33% VanI in comparison to 78%, 73% and 60% in the respective controls).

### *Ulva* zoospore germination response to synthetic AHLs

**Figure 3:**
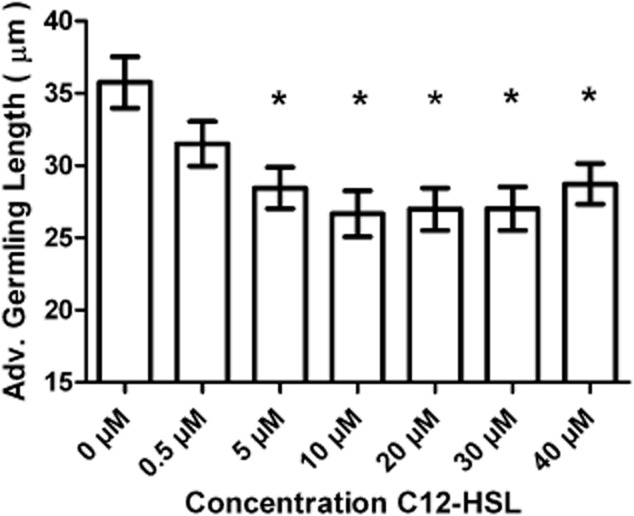
Effect of exogenously added synthetic C12-HSL on *Ulva* germling growth. Average *Ulva* germling length when exposed to a range of C12-HSL concentrations at 48 h incubation. Error bars represent 95% confidence intervals and asterisks show those values that differed significantly from that of 0 μM AHL (one-way ANOVA * = *P* ≤ 0.001).

To ensure that the effect on zoospore germination observed by AHL-producing bacteria was not just due to phenotypic changes in these bacteria, the impact of synthetic AHLs on germination was tested. The alkaline pH of seawater is known to hydrolyse the homoserine lactone ring rendering AHLs biologically inactive, and this effect is particularly prevalent in short-chain AHLs and AHLs with 3-oxy and 3-hydroxy substitutions (Hmelo and Van Mooy, 2009). Longer chain AHLs such as C12-HSL are known to be more stable in seawater (Tait *et al*., 2005; Tait and Havenhand, 2005). As AHLs with acyl side chains of 12 carbons in length are produced by both *Sulfitobacter* spp. 376 and *Shewanella* sp. 79 (Table S1), synthetic C12-HSL was tested for its effect on zoospore germination and growth at concentrations ranging from 0.5 μM to 40 μM. After 48 h incubation, there was a significant reduction in average germling length in the presence of C12-HSL concentrations ranging between 5 μM and 40 μM in comparison to the absence of C12-HSL (Fig. 3). There was also a reduction in successful zoospore germination in the presence of C12-HSL at all concentrations tested (e.g. 76% at 5 μM C12-HSL) compared with the absence of this signal molecule (86%). Furthermore, after 48 h, *Ulva* germlings exposed to synthetic C4-HSL, (an AHL also shown to be produced by both *Sulfitobacter* spp.376 and *Shewanella* spp. 79), showed a significant reduction in size and percentage of germination in the presence (26.86 μm ± 1.3 μm and 61%) compared with the absence of 5 μM C4-HSL (39.65 μm ± 1.6 μm and 86%).

## Discussion

*Ulva* zoospores preferentially settle on AHL-producing bacterial biofilms and on agarose slides permeated with synthetic AHLs (Joint *et al*., 2002; Tait *et al*., 2005) and bacteria have been shown to affect the growth of *Ulva* (Matsuo *et al*., 2003). The present study has shown that many bacteria associated with the *Ulva* thallus are AHL producers, and hence, it would be a reasonable assumption that AHLs or the products of AHL-regulated bacterial genes may also enhance *Ulva* zoospore germination and germling growth. This is the case in higher plants where AHLs are known to promote post-embryonic root development in *Arabidopsis thaliana* (Ortíz-Castro *et al*., 2008; von Rad *et al*., 2008) and can accelerate auxin-dependent adventitious root formation in *Vigna radiata* (mung bean) seedlings (Bai *et al*., 2012). In contrast, this study has shown that *U. linza* zoospore germination and the early growth of the *U. linza* germling are reduced when an AHL-producing biofilm or a synthetic source of AHLs are present. This presents a paradox; why do AHLs promote zoospore settlement but reduce zoospore germination and growth? A study of estuarine waters has shown that slower growing algal species have the ability to outcompete fast-growing species in the long term as their slow growth imposes less of a metabolic burden when nutrient conditions become limiting (Pedersen and Borum, 1996). Previous research has also shown that *Ulva spp.* are particularly sensitive to levels of nitrates and phosphates, with both nutrients shown to significantly affect the early growth of *Ulva* (Sousa *et al*., 2007). Additionally, *Ulva* growth has been shown to be dependent on salinity and light with increased light levels and a salinity of 25 psu being optimum for early *Ulva* growth (Imchen, 2012). It may be the case that in its early growth stages *Ulva* utilizes AHL signal molecules as a cue to slow its growth rate. This may lessen the rate at which *Ulva* utilizes the nutrients available in the local environment and increase the time for *Ulva* to react to local physical conditions therefore helping to facilitate survival. Retarded growth, as a result of bacterial AHL production, may also allow *Ulva* to take advantage of any extracellular growth factors bacteria provide which lead to cellular differentiation and a healthy alga. Differences in algal morphology were not observed in *Ulva* zoospores germinated with or without the presence of AHLs, and the experiments presented here were restricted to investigating the effects of AHLs on both germination and the initial germling growth period. Therefore, further research investigating the latter stages of germling growth and differentiation is required to support the hypotheses that AHLs reduce *Ulva* growth either to mediate a better chance of long-term survival and/or the successful differentiation into an adult alga. Experiments into *Ulva* germination and early growth varying both physical conditions and the presence of AHL signal molecules would also allow further testing of this hypothesis.

This investigation and studies by Tait and colleagues (2005; 2009) have demonstrated that *Ulva* zoospore germlings are constantly exposed to a variety of AHL-producing bacteria in the natural environment. Tait and colleagues (2009) provide an estimate of the concentration of AHLs within natural rocky shore biofilms, which was around 6 μM. However, natural fluctuation in the concentration of AHLs, either due to the pH-dependent hydrolysis of the homoserine lactone ring, dilution by the action of waves on the rocky shore or the production of AHL inactivating enzymes by marine bacteria such as *Shewanella* sp. (Tait *et al*., 2009), would be expected to influence *Ulva* zoospore settlement, and may also affect the germination of *Ulva* zoospores and early germling growth. Interestingly, this study observed a lack of a dose response to increasing concentrations of C12-HSL. It is thought that *Ulva* responds to AHLs via some form of yet to be discovered receptor (Wheeler *et al*., 2006; Joint *et al*., 2007). Concentrations above 5 μM C12-HSL may be saturating this receptor therefore accounting for the lack of dose response to increasing, ecologically relevant, C12-HSL concentrations. To test this further repeated experiments would have to be carried out using AHL concentrations between 0.5 μM where no significant effect was observed and 5 μM where a significant reduction in zoospore germination and germling growth was observed. Indeed, further work mimicking natural conditions are required to fully appreciate the importance of AHL signalling to both *Ulva* zoospore settlement and successful germination and growth.

Many of the bacteria isolated from the *Ulva* thallus and/or representing the *Ulva* bacterial population are actively producing a range of AHL signal molecules (Table S1). AHL production by bacterial species isolated from the marine environment and specifically from *Ulva* has been reported previously (Gram *et al*., 2002; Mohamed *et al*., 2008; Tait *et al*., 2009). The strains assayed in this study were shown to predominantly produce AHLs with shorter fatty acid side chains. The production of AHLs with short to medium length fatty acid side chains differs from previous work that has investigated AHL production in bacterial strains, isolated from a variety of marine environments, which show a predominant bias to long-chain AHL production (Gram *et al*., 2002; Taylor *et al*., 2004; Wagner-Döbler *et al*., 2005; Tait *et al*., 2009). This study used an LC-MS/MS methodology better adapted for identifying shorter chain AHLs that may not have been used in previous studies investigating marine bacterial AHL production. This may account for why we observed more bacterial strains producing these short-chain AHLs. Our findings may also indicate that short-chain AHL production is more widespread in the marine environment than suggested in previous studies. One interesting finding was C4-HSL production in strains P13, UI13 and UI08 all of which were identified as being members of the *Bacteroidetes* phylum. Until recently, AHL production was thought to be limited to Proteobacterial species. However, Romero and colleagues (2010) have equivocally demonstrated that *Tenacibaculum maritimum*, also a member of the *Bacteroidetes* phylum, produces C4-HSL, and Sharif and colleagues (2008) have also shown AHL production in cyanobacterial strains (Sharif *et al*., 2008; Romero *et al*., 2010). C4-HSL production by *Bacteroidetes* strains shown here further extends the range of species known to be undertaking AHL signal molecule production, strengthening the evidence that AHL signal molecule production is not restricted to Proteobacterial species. This study has shown the *Bacteroidetes* to be a dominant group of bacteria within the *Ulva* thallus epiphytic population and that AHL signal molecule production by these bacteria could affect the *Ulva* growth process.

Further knowledge of how AHLs and *Ulva*'s indigenous epiphytic bacterial community modulate algal growth may lead to improvements in the prevention of *Ulva* biofouling, enabling the marine industry to move away from the use of harmful antifouling agents, which can detrimentally impact the marine environment.

## Experimental Procedures

### Strains, plasmids and media

Non-marine bacterial strains and plasmids used in this study can be found in Tables S2 and S3. Post isolation marine bacterial strains were routinely cultured in Marine Broth (*Difco*, BD, Oxford, UK) at 30°C. *E. coli* strains were cultured in Luria broth (Bacto-tryptone 10 g l^−1^; Bacto yeast extract 5 g l^−1^; NaCl 10 g l^−1^) supplemented with appropriate antibiotics at 37°C (Table S3).

### Isolation and phylogenetic characterization of epiphytic bacteria

Marine bacteria were isolated from rocks colonized by *Ulva*, the *Ulva* holdfast–rock interface and from the thallus of wild *Ulva*. *Ulva* bacterial isolates were all obtained from either Wembury beach, Devon, UK (50^°^19'00' 'N 4^°^05'03' 'W), or Polzeath beach, Cornwall, UK (50^°^34'39' 'N 4^°^55'03' 'W). Bacterial isolation techniques were adapted from Patel and colleagues (2003) and Tait and colleagues (2009). Isolation of strains from the rocks colonized by *Ulva* was achieved by taking scrapings from the rocks, plating onto seawater agar (filtered seawater and 1.5% *Oxoid* number 1 agar) and incubating for 15 days at 15°C. Single colonies were then isolated on marine broth agar. Isolates from the *Ulva* thallus were obtained by vortexing *Ulva* tissue in phosphate-buffered saline (PBS) for approximately 3 min. The PBS supernatant was removed, serially diluted, plated onto either seawater agar, marine broth agar (*Difco*), Actinomycete Isolation Agar (*Difco*) or R2A agar (*Difco*) and cultured for up to 3 weeks, before being isolated on marine broth agar (*Difco*). Strains isolated from the *Ulva* holdfast–rock interface were obtained by swabbing the interface onto marine broth agar (*Difco*) and incubating for 72 h at 22°C. Single colonies were picked, streaked on marine broth agar (*Difco*) and incubated at 22°C (Patel *et al*., 2003; Tait *et al*., 2009). Phylogenetic typing of marine bacterial strains was carried out by amplifying 16S rDNA using primers 96bfm (5'-GAGTTTGATYHTGGCTCAG-3') and 1152uR (5'-ACGGHTACCTTGTTACGACTT-3') (Muhling *et al*., 2008) via colony polymerase chain reaction (PCR). PCR amplification was carried out using GoTaq DNA Polymerase (*Promega*, Southampton, UK). Reactions were made as per manufactures instructions to a total volume of 50 μl using 1 μl of boiled bacterial colony suspended in d.H_2_O has a template. Amplification was carried out using an initial step of 96°C for 2 min followed by 35 cycles of 95°C for 1 min, 53°C for 30 s and 72°C for 2 min. Amplified DNA was subsequently sequenced bidirectional using the BigDye Terminator v3.1 Cycle Sequencing Kit (Applied Biosystems, Warrington, UK) with the same primers. All sequences were submitted to GenBank (see Table S1 for accession numbers).

### DNA extraction, clone library construction and sequencing

Epiphytic bacteria were obtained from the surfaces of *Ulva* thallus material collected from Wembury beach, UK (50^°^19'00' 'N 4^°^05'03' 'W) by prolonged vortexing in sterile PBS. Bacteria were pelleted by centrifugation and DNA extracted using a Wizard DNA Purification Kit (*Promega*) as per manufacturer's instructions. 16S rDNA corresponding to nucleotides 341–926 of the *E. coli* 16S rDNA sequence was amplified via PCR from this chromosomal DNA using primers 341F (5'-CCTACGGGAGGCAGCAG-3') and 907R (5'-CCGTCAATTCMTTTGAGTTT-3'). PCR was carried out using the same condition described previously with the exception of the extension time which was 1 min. The amplified 16S rDNA was cloned into the pGEM T easy vector (*Promega*) and transformed into *E. coli* DH5a via electroporation according to the manufacturer's instructions. A total of 96 clones were selected for sequencing using the M13F and M13R primers. Sequencing was bidirectional using the BigDye Terminator v3.1 Cycle Sequencing Kit (Applied Biosystems). Phylogenetic analysis and tree topology was carried out using the MEGA programme (Tamura *et al*., 2011).

### AHL extraction, detection and characterization assays

AHLs were extracted from 20 ml of cell-free culture supernatants acidified with 2 M HCl to pH 2 using dichloromethane in accordance with the method described by Yates and colleagues (2002). AHL detection assays were carried in accordance with the method described by Tait and colleagues (2009) using bioreporters *E. coli* JM109 pSB536, pSB401 and pSB1075 (Swift *et al*., 1997; Winson *et al*., 1998). A black clear bottomed 96-well microtitre plate (*Greiner Bio One*, Gloucestershire, UK) was used for all assays with 100 μl of cell-free supernatant extract dried to each well assayed. After 3 h coincubation with bioreporters at 37°C, the resulting luminescence was measured using a *Berthold* MITHRAS microtitre plate reader (*Berthold*, Bad Wildbad, Germany). Intensity of bioluminescence was calculated in relative light units (Tait *et al*., 2009). AHLs from extracted planktonic cultures were further characterized by LC-MS/MS using a methodology previously described by Yates and colleagues (2002). Solvent extracts were separated by reverse phase high performance liquid chromatography using and Ascentis Express C18 2.7 μm column (150 × 2.1 mm) coupled to a tandem mass spectrometer (Bruker HCT Plus ion trap; Bruker Daltoniks, Bremen, Germany). Extracted ion chromatograms of precursor ion *m/z* 102.1 (homoserine lactone ring) were produced from the mass of the parent molecular ion [M + H] of each AHL screened for, (for fragment ion *m/z* of each AHL, see Yates *et al*., 2002), and retention times and peak spectra were matched to 1 μM standards of each AHL (Yates *et al*., 2002; Ortori *et al*., 2007; 2011).

### Growth of biofilm material

Bacterial biofilms were grown from varying volumes of 0.5 ml, 1 ml, 2 ml and 4 ml initial inoculum in accordance with methods described by Tait and colleagues (2005). Biofilm inoculum was prepared using stationary phase cultures which were pelleted, washed in sterile filtered seawater and adjusted to an OD_600_ of 1.00 before inoculation. Marine biofilms were grown for 72 h at 22°C. Biofilms of *E. coli* strains were grown in 70% filtered seawater for 24 h in order to reduce osmotic stress on the bacteria by salinity (Tait *et al*., 2005). Biofilm density was determined as per the method described by Joint and colleagues (2002) with microscope image analysis at 400× magnification, using a Reichert Jung Polyvar microscope (Leica microsystems, Milton Keynes, UK) and an Optronics Magna Fire SP camera (Milton Keynes, UK) (Joint *et al*., 2002).

### *Ulva* zoospore germination assays

*Ulva* zoospore release was carried out in accordance with methodology of Callow and colleagues (1997). All *Ulva* zoospores used in this study were released from fertile *Ulva* thalli collected from Wembury beach, UK (50^°^19'00' 'N 4^°^05'03' 'W) (Callow *et al*., 1997). *Ulva* zoospores were settled onto biofilms or sterile glass slides using the method described by Tait and colleagues (2005); in all cases, 15 ml of the final *Ulva* zoospore suspension adjusted to an OD_600_ of 0.5 was used; additionally, all experimental treatments were carried out on three separate slides. Post settlement zoospore slides were transferred to sterile 60 mm Petri dishes and submerged in 10 ml sterile filtered seawater. For zoospore assays using *E. coli* biofilms, 70% sterile filtered seawater was used. For the assays using synthetic AHLs, C12-HSL was added to the separate dishes post zoospore settlement at final concentrations ranging from 0.5 μM and 40 μM and at 5 μM for C4-HSL. Zoospore slides were incubated at 18°C in proximity to a light source with a 16 h light, 8 h dark cycle for 48 h (24 h for assay using *E. coli* biofilms). After incubation, the zoospore slides were fixed with 2% (v/v) glutaraldehyde and stained with dilute carbol fuchsin.

### Image and statistical analysis

For all *Ulva* zoospore germination assays, the three separate zoospore slides for each experimental treatment were viewed at 100× magnification using a Reichert Jung Polyvar microscope with attached Optronics Magna Fire SP camera. The zoospore slides from each experimental condition were imaged 10 times in randomly selected locations. The lengths of 300 individual *Ulva* zoospores and germlings were measured from randomly selected images of each experimental condition using Image ProPlus Version 5 imaging software (Media Cybernetics, Rockville, MD, USA). Zoospores over 15 μm were defined as successfully germinated, as such percentage zoospore germination was calculated by dividing the number of successfully germinated zoospores by the total number of settled zoospores. Statistical analysis of *Ulva* zoospore germination assays was carried out by performing analysis of variance (ANOVA) tests using Minitab 16 (*Minitab*).

## Supporting Information

**Fig. S1.** Phylogenetic tree of the *Ulva* thallus bacterial population**.** The tree resulted from a sequence alignment of 16S rDNA from bacterial clones and isolates obtained from the *Ulva* thallus at 97% sequence similarity. The reference strains were taken from GenBank. In brackets is the number of clones in each OTU. The tree topology is based on neighbour- joining and bootstrap analysis was performed with 1000 replications.

**Fig. S2.** AHL bioreporter activation assay. A. Cell-free supernatant extracts from *Shewanella* spp. 79 pBBRIMCS and pMT01 were assayed with *lux*-based AHL bioreporters *E. coli* pSB536 (clear bars) and pSB1075 (cross-hatched bars). B. Cell-free supernatant extracts from *Sulfitobacter* spp. 376 pBBRIMCS and pMT01 were assayed with *lux*-based AHL bioreporter *E. coli* pSB401. All strains harbouring pMT01 and therefore expressing the *aiiA* lactonase failed to activate the bioreporters showing that their cognate AHLs were being deactivated.

**Fig. S3.** AHL germination assays slides. Example images of *Ulva* zoospores germinated on (A) *Sulfitobacter sp.* pMT01 biofilms (AHL-deficient) and (B) *Sulfitobacter sp.* pBBRIMCS biofilms (AHL-producing). Bar = 50 μm.

**Table S1.** Phylogenetic identification and AHL signal molecule production in *Ulva*-associated marine bacteria. The ability of each strain to produce AHLs was assayed using *lux*-based AHL bioreporters, a positive result is indicated by **+** and a negative result by **–**. AHL production and identity was confirmed by LC-MS/MS. **Table S2.** Non-marine bacterial strains used in this study. **Table S3.** Plasmids used in this study.
