# Supplementary material for: Thematic Issue: Protists
Source: Environ Microbiol. 2013 Jul 23;16(2):445–53. doi: 10.1111/emi.12203 (PMC4304359; doi:10.1111/emi.12203)
Supplement: Supplementary file 4 — Table S1. Phylogenetic identification and AHL signal molecule production in Ulva-associated marine bacteria. The ability of each strain to produce AHLs was assayed using lux-based AHL bioreporters, a positive result is indicated by + and a negative result by –. AHL production and identity was confirmed by LC-MS/MS. Table S2. Non-marine bacterial strains used in this study. Table S3. Plasmids used in this study. [file emi0016-0445-SD4.docx]

Supplementary Material

Interference with the germination and growth of Ulva zoospores by quorum sensing molecules from Ulva-associated epiphytic bacteria

Matthew S. Twigg, ^1, 2^ Karen Tait, ^2*^, Paul Williams^1^, Steve Atkinson^1^ and Miguel Cámara^1^

^1^School of Molecular Medical Sciences, Centre for Biomedical Sciences, University of Nottingham, NG7 2RD, UK.

^2^Plymouth Marine Laboratory, Prospect Place, Plymouth, PL1 3DH, UK.

*For correspondence

Email: ktait@pml.ac.uk

Phone Number: +44(0)1752 633100

Fax Number: +44(0)1752 633101

Legends to Supplementary Figures

Supplementary Figure 1

**Phylogenetic tree of the Ulva thallus bacterial population.** The tree resulted from a sequence alignment of 16S rDNA from bacterial clones and isolates obtained from the Ulva thallus at 97% sequence similarity. The reference strains were taken from Genbank. In brackets is the number of clones in each OTU. The tree topology is based on neighbour- joining and bootstrap analysis was performed with 1000 replications.

Supplementary Figure 2

AHL bio-reporter activation assay. (A) Cell free supernatant extracts from Shewanella spp. 79 pBBRIMCS and pMT01 were assayed with *lux*-based AHL bio-reporters *E. coli* pSB536 (clear bars) and pSB1075 (cross-hatched bars). **(B)** Cell free supernatant extracts from Sulfitobacter spp. 376 pBBRIMCS and pMT01 were assayed with *lux*-based AHL bio-reporter *E. coli* pSB401. All strains harbouring pMT01 and therefore expressing the *aiiA* lactonase failed to activate the bio-reporters showing that their cognate AHLs were being de-activated.

**Supplementary Figure 3**

**AHL Germination Assays Slides.** Example images of *Ulva* zoospores germinated on (**A**) *Sulfitobacter sp.* pMT01 biofilms (AHL- deficient) and (**B**) *Sulfitobacter sp.* pBBRIMCS biofilms (AHL-producing). Bar = 50 µm.

Supplementary Table 1. Phylogenetic identification and AHL signal molecule production in Ulva associated marine bacteria. The ability of each strain to produce AHLs was assayed using lux-based AHL bio-reporters, a positive result is indicated by + and a negative result by -. AHL production and identity was confirmed by LC-MS/MS.

|  | | |  |  | | | **AHL Production** | | | |
| --- | --- | --- | --- | --- | --- | --- | --- | --- | --- | --- |
|  |  |  | | |  |  | **Bio-reporter Assay** | | |  |
| **Strain** | **Accession Num.** | **Identification** | | | **Phylogeny** | **Sample** | **pSB536** | **pSB401** | **pSB1075** | **LC-MS/MS** |
| UI08 | KC592366 | *Cellulophaga* sp. | | | *Bacteroidetes* | *Ulva* thallus | **+** | **-** | **-** | C4-HSL |
| 243 | KC608161 | *Roseobacter* sp. | | | *Alphaproteobacteria* | Rocky shore | **+** | **-** | **-** | C4-HSL |
| P13 | KC592372 | *Flavobacterium* sp. | | | *Bacteroidetes* | Rocky shore | **+** | **-** | **-** | C4-HSL |
| UI13 | KC592367 | *Cellulophaga* sp. | | | *Bacteroidetes* | *Ulva* thallus | **+** | **-** | **-** | C4-HSL |
| UI20 | KC592370 | *Pseudomonas* sp. | | | *Gammaproteobacteria* | *Ulva* thallus | **+** | **-** | **-** | C4-HSL, C8-HSL |
| 371 | KC592374 | *Sulfitobacter* sp. | | | *Alphaproteobacteria* | Rocky shore | **+** | **-** | **+** | C4-HSL, 3-oxo-C8-HSL, 3-oxo-C12-HSL |
| UI19 | KC592369 | *Alteromonas* sp. | | | *Gammaproteobacteria* | *Ulva* thallus | **+** | **+** | **-** | C4-HSL |
| UI33 | KC592368 | *Marinobacter* sp. | | | *Gammaproteobacteria* | *Ulva* thallus | **+** | **-** | **-** | C4-HSL, 3-OH-C8-HSL |
| 79 | KC592373 | *Shewanella* sp. | | | *Gammaproteobacteria* | *Ulva* thallus | **+** | **-** | **+** | C4-HSL, C12-HSL |
| RUBI03 | KC592371 | *Paracoccus* sp*.* | | | *Alphaproteobacteria* | *Ulva* holdfast | **+** | **+** | **-** | C4-HSL, 3-oxo-C8-HSL, C14-HSL |
| 376 | KC608162 | *Sulfitobacter* sp. | | | *Alphaproteobacteria* | Rocky shore | **-** | **+** | **-** | C4-HSL, 3-oxo-C8-HSL, 3-oxo-C12-HSL |

Supplementary Table 2. Non marine bacterial strain used in this study

| Strain | Genotype | Reference |
| --- | --- | --- |
| E. coli |  |  |
| DH5α | sup#794 ΔlacU169 (Φ80 lacZ ΔM15) hsdR17 recA1 endA1 gyrA96 thi-1 relA1 | (Sambrook and Russell, 2001) |
| JM109 | recA1 sup#794 endA1 hsdR17 gypA96 relA1 thi D (lac-proAB) | (Yanisch-Perron et al., 1985) |

Sambrook, J., and Russell, D.W. (2001) *Molecular Cloning: A Laboratory Manual*, 3rd Edn. Cold Springs Harbor, USA:

Cold Springs Harbor Laboratory Press.

Supplementary Table 3. Plasmids used in this study

| Plasmid | Description | Reference/ Source |
| --- | --- | --- |
| pGEM T Easy | Cloning vector with an f1 origin of replication containing lacZ, Amp^r^ and a multiple cloning site. | Promega |
| pBBRIMCS-1 | Broad host range vector containing lacZ, Cm^r^ and a multiple cloning site. | (Kovach et al., 1994) |
| pSB536 | AHL bio-reporter composed of a pUC18 derived plasmid containing Amp^r^ and a fusion of AyhR and lux promotor from Vibrio fischeri to the lux operon from Photorhabdus luminescens. | (Swift et al., 1997) |
| pSB401 | AHL bio-reporter composed of a pACYC184 derived plasmid containing Tet^r^ and a fusion of luxR and lux promotor from Vibrio fischeri to the lux operon from Photorhabdus luminescens. | (Winson et al., 1998) |
| pSB1075 | AHL bio-reporter composed of a pACYC184 derived plasmid containing Tet^r^ and a fusion of lasR and lasI promotor from Pseudomonas aeruginosa to the lux operon from Photorhabdus luminescens. | (Winson et al., 1998) |
| pBBRIMCS-5 | Broad range cloning vector | (Kovach et al., 1995) |
| pMT01 | pBBRIMCS-5 vector containing aiiA from pSU18::aiiA as an EcoRI fragment | This study |
| pT7T3 | Cloning vector used as wt control in E. coli Ulva zoospore germination assay | (Tait et al., 2005) |
| pET3a | Cloning vector used as wt control in E. coli Ulva zoospore germination assay | (Wheeler et al., 2006) |
| pT7T3luxI | Plasmid expressing Vibrio fischeri AHL synthase gene luxI | (Tait et al., 2005) |
| pMW47.1 | Plasmid expressing Pseudomonas aeruginosa AHL synthase gene *rhlI* | (Latifi et al., 1995) |
| pETVanI2 | Plasmid expressing Vibrio anguillarum AHL synthase gene vanI | (Tait et al., 2005) |

Kovach, M.E., Phillips, R.W., Elzer, P.H., Roop, R.M., 2nd,and Peterson, K.M. (1994) pBBR1MCS: a broad-hostrange

cloning vector. *Biotechniques* **16:** 800–802.
